# 2198 Cognitive and behavioral side effects in patients treated with droxidopa for neurogenic orthostatic hypotension

**DOI:** 10.1017/cts.2018.158

**Published:** 2018-11-21

**Authors:** Katherine McDonell, Cyndya Shibao, Italo Biaggioni, David Robertson, Daniel Claassen

**Affiliations:** Vanderbilt University Medical Center

## Abstract

OBJECTIVES/SPECIFIC AIMS: To describe adverse behavioral symptoms attributed to droxidopa therapy for neurogenic orthostatic hypotension (nOH). METHODS/STUDY POPULATION: BACKGROUND: Droxidopa, a norepinephrine (NE) precursor, improves symptoms of nOH by replenishing NE levels. Central NE effects are poorly described but may offer potential benefits given the pathophysiologic progression of α-synuclein-related disorders. Here we report a series of cognitive and behavioral side effects linked to droxidopa therapy. METHODS: We identified 5 patients treated at Vanderbilt University who developed behavioral symptoms including mania, irritability, and disorientation shortly after the initiation of droxidopa for nOH. Comprehensive chart reviews were performed for all patients, including analysis of droxidopa titration schedule and dosing, medical comorbidities, clinical course, and outcome. All patients had symptoms of synucleinopathy, manifesting with autonomic failure, REM behavior disorder, and parkinsonism. Four met criteria for idiopathic PD, and one was diagnosed with pure autonomic failure but had concomitant symptoms of parkinsonism and REM sleep behavior disorder. RESULTS/ANTICIPATED RESULTS: Our patients had no significant cognitive or behavioral symptoms before the initiation of droxidopa. The average decrease in blood pressure upon standing was 27 mmHg systolic and 17 mmHg diastolic. Behavioral disturbances were observed early in the titration period and at relatively low doses of droxidopa (total daily doses ranging from 300 to 800 mg/day; droxidopa therapeutic dose range 900–1800 mg/d). The most common symptoms reported were mania, irritability, and confusion. Symptoms resolved with dose reduction in 4 patients, and droxidopa was discontinued in 1 patient due to persistent irritability. No other medical comorbidities or alternative etiologies were identified to explain these effects. DISCUSSION/SIGNIFICANCE OF IMPACT: Droxidopa is a prodrug designed to act peripherally, but may also have important, yet poorly described, central effects. We hypothesize that these behavioral manifestations result from an “overdose” of key NE networks linking orbitofrontal and mesolimbic regions. Further studies are warranted to better characterize central NE effects in patients treated with droxidopa.

